# The role of power in health policy dialogues: lessons from African countries

**DOI:** 10.1186/s12913-016-1456-9

**Published:** 2016-07-18

**Authors:** Aziza Mwisongo, Juliet Nabyonga-Orem, Theodore Yao, Delanyo Dovlo

**Affiliations:** 1 Health Systems and Services Cluster, World Health Organization Regional Office for Africa, B.P. 06, Brazzaville, Congo; 2 Health Systems and Services, World Health Organization Country Office, Boîte postale 99, Bamako, Mali

**Keywords:** Policy dialogue, Policy development, Power, Low income countries

## Abstract

**Background:**

Policy-making is a dynamic process involving the interplay of various factors. Power and its role are some of its core components. Though power exerts a profound role in policy-making, empirical evidence suggests that health policy analysis has paid only limited attention to the role of power, particularly in policy dialogues.

**Methods:**

This exploratory study, which used qualitative methods, had the main aim of learning about and understanding policy dialogues in five African countries and how power influences such processes. Data were collected using key informant interviews. An interview guide was developed with standardised questions and probes on the policy dialogues in each country. This paper utilises these data plus document review to understand how power was manifested during the policy dialogues. Reference is made to the Arts and Tatenhove conceptual framework on power dimensions to understand how power featured during the policy dialogues in African health contexts. Arts and Tatenhove conceptualise power in policy-making in relational, dispositional and structural layers.

**Results:**

Our study found that power was applied positively during the dialogues to prioritise agendas, fast-track processes, reorganise positions, focus attention on certain items and foster involvement of the community. Power was applied negatively during the dialogues, for example when position was used to control and shape dialogues, which limited innovation, and when knowledge power was used to influence decisions and the direction of the dialogues. Transitive power was used to challenge the government to think of implementation issues often forgotten during policy-making processes. Dispositional power was the most complex form of power expressed both overtly and covertly. Structural power was manifested socially, culturally, politically, legally and economically.

**Conclusions:**

This study shows that we need to be cognisant of the role of power during policy dialogues and put mechanisms in place to manage its influence. There is need for more research to determine how to channel power influence policy-making processes positively, for example through interactive policy dialogues.

## Background

Policy-making is a dynamic process involving the interplay of various factors, with power, one of its core components, at the heart of every policy process [1]. Fundamental to any policy process are the roles of the actors. The actors influence the process through their knowledge, experiences, beliefs and power [1]. Although power has a profound role in policy-making, empirical evidence suggests that health policy analysis has paid only limited attention to the issue of power in low and middle income countries [2]. Further, power is rarely examined because this is difficult, requiring a combination of empirical evidence and theorising. The definition of power is ambiguous too, especially when it is used in operational terms for research [3]. Dhal [3] states that the way power is defined and understood has implications in policy-making. There are conflicting views on power dynamics, including on the common assumption that power is and should always be top-down.

Many of the debates around policy processes characterise them as top-down with little need for the involvement of the ground level implementers. A consequence of this traditional policy-making approach is that many policies are poorly implemented vis-à-vis their objectives [1, 4, 5]. Furthermore, the probability of a prescribed policy being disputed is much higher if the implementers are not involved in its making [5]. Policy implementers tend to lie at the bottom end of the policy-making spectrum and their involvement in policy discussions is generally limited [5–7]. This has led experts in policy to suggest the use of other approaches to policy-making and implementation such as the *top*-*bottom*-*bottom*-*top* strategies and the *bottom*-*up* approach. Such approaches have proved to be significant in enhancing policy-making and implementation, as seen, for example, through the adoption of the World Health Organization’s (WHO) Framework Convention on Tobacco Control, which established a precedent for worldwide actions targeting the supply and demand feedback on tobacco use [8]. This bottom up approach to policy-making is also commonly used outside the health sector, with positive outcomes [4].

Lately there has been a move towards interactive and more participatory approaches to policy-making [9, 10]. Some of the new concepts underlying this approach include interactive policy-making and planning, network management, stakeholder dialogue, deliberative democracy, policy discourses and governance [4]. Policy dialogues are highly recommended as a means of achieving interactive and inclusive policy-making, but studies conclude that they are only valuable if they are well conducted, participatory and evidenced informed [11–14]. The supporters of the paradigm shift in policy-making argue for more collective engagement of stakeholders. However, power cannot be ignored even in such interactive processes. In real life power is manifested through multiple ways like resources, capacity and knowledge [3, 4, 15]. Health policy analysts contend that power still has a role to play in policy-making and should not be ignored [1, 4, 15]. The literature says that power influences policy processes and outcomes in a multitude of ways, such as through the relationships between actors, trust and the manner in which policy-makers act with tactical exclusions of certain issues or people [1, 4, 15]. Their arguments are derived from various theories of power that show how it influences actors’ organisational behaviour and positions in policy-making. Nevertheless there is still limited examination of how power manifest among actors in different contexts in relation to policy making.

In 2011 the European Union, WHO and the Government of Luxembourg entered into a collaborative agreement to support policy dialogue on national health policies, strategies and plans. Referred to as the EU-Lux-WHO Policy Dialogue Programme, the ultimate aim of this collaboration was to improve health sector outcomes in targeted countries, with an overall focus on promoting universal health coverage (UHC), people-centred health care and inclusion of health in all policies. More specifically, the policy dialogue programme aimed to build the capacities of the participating countries to develop, negotiate, implement, monitor and evaluate evidence-based and all-inclusive national health policies, strategies and plans. This set a precedent to a number of policy dialogues in some African countries.

Policy dialogue in health is a new area of research, as reflected by the paucity of evidence in the main literatures. This paper thus uses qualitative data on policy dialogues from Chad, Cabo Verde, Guinea, Liberia and Togo to understand the different forms of power and its expression in policy-making. Looking at how power was manifested in these dialogues is useful for policy-makers, researchers and others. For policy-makers it is important to know how power played out in the dialogues to understand their outcomes and how to manage power better within such dialogues in the future. The paper utilises the power model developed by Art and Tatenhove [4], which depends heavily on the policy arrangement approach combined with different power theories. The model is used to study and comprehend the dimensions of power and the consequences of its use during policy dialogues. Given that theoretical models on power have not been used in policy-making before, this paper will contribute to understanding of power in the policy analysis arena. The Art and Tatenhove framework is useful, as it specifically applies to understanding of the different dimensions of power.

There is paucity of literature on power and policy-making. In this study we refer to the Arts and Tatenhove [4] conceptual framework on power dimensions to understand how power features and influences policy dialogues. Arts and Tatenhove conceptualise power in policy-making to have three layers (Table 1). They argue that policy-making is a dynamic process, that power is central to this process and that power is inevitable and is often exercised in any kind of relationship.

**Table 1:** The three layers of power in policy-making

Type of power	Focus	Policy concept
Relational (transitive and intransitive)	Achievement of policy outcomes by agents’ interaction	Policy innovation
Dispositional	Positioning of agents in arrangements mediated by rules and resources	Policy arrangements
Structural	Structuring of arrangements mediated by orders of signification, domination and legitimization	Political modernization

Source: Arts B, Van Tatenhove J. Policy and power: A conceptual framework between the ‘old’ and ‘new’ policy idioms. Policy Sciences 2004;37 (3–4):339–356

*Relational power* relates to the dynamics that exist among actors, resources, outcomes and interactions [4]. To Art and Tatenhove, the relational concept of power is based on the belief that in any relationship there exists some form of dominance [4]. There are two forms of relational power: transitive and intransitive. Transitive relational power, also known as a *zero*-*sum game*, concerns situations where a single group achieves certain outcomes at the expense of others. Intransitive power is concerned more with collective bargaining of a community to achieve a certain goal.

*Dispositional power* refers to the shaping of the power of agents by their organisations’ rules and resources [4]. This type of power results from agents being placed in certain positions that make them more influential than the others [4]. Art and Tatenhove state that the positioning of the agents inevitably renders them the legitimacy, authority and ability to allocate resources [4].

With *structural power*, both individual and organisational conduct are shaped by existing micro-societal structures, which are governed by signification, legitimisation and domination [4].

## Methods

### Study design

This was an exploratory and descriptive study that utilised qualitative methods. The main aim was to explore and understand the experiences in policy dialogues and how power impacted such processes in five African countries. The objective was to explore and understand the processes related to the actors’ involvement in policy dialogues, the factors that influenced the dialogue process and the impact of the dialogues on health sector performance outcomes and processes. Data were collected through a review of relevant government documents and interviews with key informants. Using an interview guide, we collected data from 90 key informants in Cabo Verde, Chad, Guinea, Liberia and Togo that had been engaged in country level policy dialogue processes at both the national and sub-national levels. Data collection occurred June to August 2015.

### Selection of respondents

At the national level, the initial step was to hold discussions with WHO country office and ministry of health technical officers responsible for convening the dialogue meetings, who identified the key agencies involved. At the sub-national level, the first step was to meet the head of the district health office, who identified the ministry of health officers and the agencies involved in the dialogue process. Within the identified agencies, the key informants were purposively selected based on their participation in the policy dialogue processes, seniority and knowledge on the research question [16]. Additional key informants were identified through the snowballing technique until descriptive saturation was achieved [17]. All the identified informants were invited by phone to participate in the interviews, and the 98 % of them who agreed to participate were interviewed. The details on the key informants in the five countries are shown in Table 2.

**Table 2:** Key respondents in Cabo Verde, Guinea, Liberia, Togo and Chad

Category	Cabo Verde	Chad	Guinea	Liberia	Togo
National					
Ministry of Health	5	6	15	4	8
Donor agencies	1	4	6	3	1
Civil society	6	0	3	3	4
Sub-national					
Ministry of Health	0	4	3	1	4
Donor agencies	0	0	0	0	0
Civil society	2	0	1	2	4
Total	14	14	28	13	21

### Data collection methods

The interview guide had standardised questions and probes on the interaction of actors during the policy dialogue. For each country the information sought included the dialogues’ contextualisation, governance and management, and power relations. In each country, data were collected by an independent researcher with expertise in qualitative research and knowledge of health policy and systems. The interviews were conducted in English, French or Portuguese and lasted 45 min on average.

All the audio-recorded interviews were transcribed verbatim and later translated into English by the researchers and the WHO Regional Office for Africa translation unit. The transcripts were then exported into MAXQDA software for analysis. Codes and sub-codes were developed guided by the Art and Tatenhove conceptual framework on power. These codes were compared for the key informants in each country and then across the countries.

## Results and discussion

This section presents the findings from our study married with existing evidence. It begins with the experiences in the dialogues in the five countries, followed with a detailed description of the forms of power that were exhibited within the dialogues. It ends with a section on how policy dialogues are influenced by power and context.

### Policy dialogue experiences in Chad, Cabo Verde, Guinea, Liberia and Togo

Policy dialogues in the five countries had a high level of participation and involved a wide range of actors, including representatives of the ministries of health, finance, and social services; donor partners; the civil society; unions; sub-national ministry of health sections such as regional and district health management teams; and the community. Table 3 presents details on the actors and their roles.

**Table 3:** Roles of the actors involved in the policy dialogues

Group	Actors	Roles	Interest
National level ministries	Ministers, heads of departments, technical officers	Policy-making in their specific sectors and their departments	Ensuring effective policy-making while safeguarding their roles
Donors	Technical officers	Representing their organisations and finding opportunities to support government efforts in line with their organisations’ agenda and interests	Getting involved in policy-making while also driving their organisation’s agenda
Civil society	Executive officers	Promoting good governance practices like transparency, effectiveness, openness, responsiveness and accountability	Getting involved in the policy process in order to advocate for certain rights but also to create and sustain visibility and viability
Unions	Representatives	Protecting the integrity of their trade and advocating for workers’ rights and incentives	Ensuring that workers’ rights and incentives are considered during policy-making
Sub-national ministry of health officials	Regional and district officers	Implementing programmes	Ensuring that the factors that influence programme implementation are considered during the policy-making process
Community representatives	Community leaders	Representing and safeguarding community interests	Ensuring that community interests are considered during the policy-making process

The EU-Luxembourg-WHO support for the dialogues in the countries stems from the need to strengthen UHC. The policy dialogues addressed various issues according to the countries’ needs and to support UHC initiatives. The issues related to strengthening of the planning processes, monitoring and evaluation of health programmes, health financing, and improvement of alignment and harmonisation of health stakeholders. Table 4 highlights the main policy dialogues that were conducted in the five countries with the EU-Luxembourg-WHO Policy Dialogue Programme.

**Table 4:** Types of policy dialogues conducted in Cabo Verde, Guinea, Liberia, Togo and Chad

Areas of support	Examples of policy dialogues
Development and implementation of robust national health policies, strategies and plans; increased coverage with essential health services; financial risk protection; and health equity	• Health sector investment and recovery plans in Liberia and Guinea
	• Development of the national health policies, strategies and plans in Mali, Togo and Cabo Verde
	• Evidence-based planning (resource mapping, comprehensive health situation analysis) in Liberia and Guinea
	• Strengthened sub-national capacity for planning, for example through developing inclusive operational plans, for example in Liberia and Guinea
Improvement of technical and institutional capacities, knowledge and information for adaptation by health systems and services and for related policy dialogues	• Strengthening participatory review mechanisms through joint annual reviews and evaluations of the national health policies, strategies and plans, for example in Guinea, Togo and Cabo Verde
Ensuring that international and national stakeholders are increasingly aligned around national health policies, strategies and plans and adhere to other aid effectiveness principles	• Conducting and ensuring continuous and sustained dialogues at national and sub-national levels in all the five countries

In each country the policy dialogues occurred at various levels, starting from the community level. Broadly, the dialogues can be categorised as follows:

- Community level policy dialogues were mainly organised by nongovernmental organisations (NGOs). The information obtained from these dialogues fed into either the district or regional level dialogues, but sometimes it was presented directly by the NGOs or community representatives at national level dialogues.
- District level dialogues’ outcomes were fed into regional dialogues and sometimes directly into national level dialogues. In some instances these dialogues were organised according to the specific areas of focus such as diseases like Ebola and HIV in Liberia and Togo, respectively.
- National level dialogues took several forms. On some occasions they began at the departmental level and then cascaded to the ministerial level for additional discussion. Where the issues were not resolved, they were moved to the larger national level meetings. Some dialogues at the national level were held by a representative group on behalf of the ministry of health, such as the pharmacy forum in Cabo Verde. In Togo, for example, there was a need at the department level to determine how the move towards UHC would be financed. This necessitated a dialogue with stakeholders to explore the options. The evidence available on the status of financing for the health sector informed the dialogues at the departmental, ministerial and national levels. The dialogues involved stakeholders from the presidency, various ministerial departments, social partners, civil society, the private sector and funding partners. Consensus was reached on the future financing options, the details of which were incorporated into the health financing strategy.

**Fig. 1:**
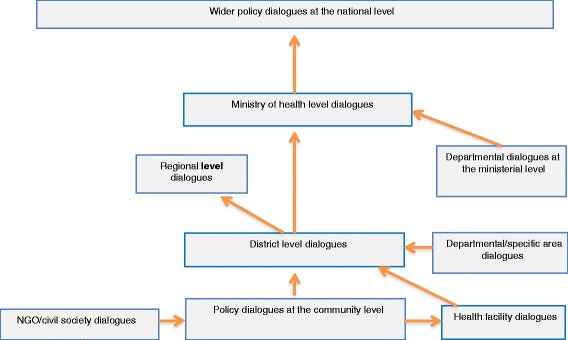
Types and logical flow of policy dialogues in Cape Verde, Guinea, Liberia, Togo and Chad

Figure 1 shows the levels at which the dialogues were conducted and how they fed into the bigger dialogues at the national level.

### Forms of power exhibited in the policy dialogues

#### Relational power

Relational power is the authority that agents use to achieve outcomes through their interactions [4]. By and large, at this level the power interplay constitutes the actors and their resources, mediated through societal interactions [4]. One type of relational power, transitive power, is concerned with winning at the expense of the other party, regardless of the fact that this is a zero-sum game (situation where one or more participants’ gain (loss) equals the loss (gain) of other participants) [4]. There are several examples from the policy dialogues where stakeholders had to succumb to pressure from the other party on a particular demand in order to ensure that the policy dialogue proceeded to achieve the intended outcomes. In Cabo Verde, for example, during the dialogues for the development of the pharmaceutical policy, tension arose between government representatives and private pharmacists regarding the sale of brand drugs, as noted by a stakeholder,

*The Ministry of Health had to agree to our request for provision of brand drugs if they wanted our support* … *after all we are doing business. This was a tough decision* (Private pharmacist, Cabo Verde).

Although government officials in Cabo Verde wanted to restrict sales of only generic drugs, they were forced to consider inclusion of brand drugs in order to develop the policy. According to the respondents, in Togo there was tension in the debates between the state and civil society organisations (mutual health organisations) over the organisation of health insurance, which forced the government into more dialogues at the sub-national level to agree on the role of insurance entities. In Liberia the handling of divergent views over the ministerial role in UHC are a good example of how relational power was applied, as noted by a respondent,

*We had so many delays because of the disagreement between the ministries of health and social affairs on their roles in UHC. Through several dialogues and presentations it became clear that it was better to place it under the Ministry of Social Affairs* … *the Ministry of Health had to agree in order to move the process* (Ministry of Health official, Liberia).

In these examples, although the pharmacists, the civil society organisations and the Ministry of Social Affairs could be regarded as the winners, in the real sense the losers, mostly the government, stood to benefit in the end because it was they who were responsible for developing the specified policies. This was a zero-sum game situation [4]. It could also be argued that although a policy is developed, it may not achieve its goal because of the power battles that have influenced it, limiting its benefit.

Some theorists argue that not all power is motivated by the need to protect personal benefits and that sometimes power is exerted and used merely to push for better performance of the health system [1]. According to the respondents, this was seen in the dialogues in Togo, during which a bottom-up approach was used to gather information and evidence from sub-national dialogues. Prescribed processes from the national level, as a basis for developing strategies, were resisted by the sub-national level and instead they used their own processes supported with evidence from their districts.

Intransitive power, which calls for unification of efforts towards a common output, was applied on several occasions during these dialogues to produce collective outcomes. From the interviews, it was evident that in all the five countries the stakeholders involved in the policy dialogues worked in a unified manner to produce a document or policy of interest. For example, in Cabo Verde, during the development of the pharmaceutical policy, the pharmacy forum and its members worked together to ensure that the policy was a success, notwithstanding the divergent views among the group members. It was clear that all the stakeholders had the one major objective to have a policy that would guide them in their pharmaceutical practices in Cabo Verde, as one respondent stated,

*We worked tirelessly to ensure that the pharmaceutical policy was completed* … *it was time to have an updated one*, *as it affected all of us* (National level official, Cabo Verde).

In Chad and Liberia, structures such as the annual health sector review meetings embracing a number of stakeholders were used as forums for policy dialogues in the development of various policies. In such instances, existing forums were effective in galvanising different stakeholders with conflicting opinions to work together towards a common outcome.

### Dispositional power

In this study, dispositional power, which mostly is influenced by agents’ position to act, was observed in several contexts. One form of dispositional power was associated with hierarchical positions inherent in administrative structures and organisations. In all the policy dialogues in the five countries the ministry of health and the district health management teams were able to spearhead the discourses owing to their positional authority or authoritative resources. Further, the ministry of health had the upper hand in the policy dialogue because of its mandate in health. In most of these countries the existing hierarchy systems and contexts of neo-patriotic governance, characterised by a strong patron-client relationships between government officials and citizens or the public, also made it easier for dispositional power to be exercised. For example, in Liberia there were many instances were specific themes were selected by the Ministry of Health with no clear justification, as one respondent noted,

*It was not quite clear how the themes were derived in these dialogues. You were invited to a dialogue on national health accounts without even knowing why and how it came about* (NGO official, Liberia).

Dispositional power was manifested also at the sub-national level, where some policy dialogues were conducted using a bottom-up approach but with the content and context mostly predetermined by the ministry of health. The sound knowledge and skills of the district health management team resulted from their ability to lead these policy dialogues. Prior to the dialogues, the team underwent training in stakeholder dialoguing. This strengthened their structural power by adding to their knowledge and skills, [9]. Literature shows that these attributes, along with professionalism, engender a certain kind of power [9]. In this context, the health management teams complemented their existing dispositional power with the new knowledge and skills, strengthening their influence in the policy dialogues that they conducted.

Whitefield and Fraser [18] use the term negotiating capital to define the negotiator’s resources associated with certain structural conditions such as economic conditions, ideological conditions and institutional conditions. Economic conditions refer to the negotiator’s degree of financial dependency. Most of the countries that were studied have a high donor dependency, and several respondents indicated that donors had a big influence in the policy dialogues. For example, in Guinea the EU-WHO joint programme had helped to spearhead the policy dialogues, but to many respondents WHO had much more control over the process than they would have expected. One of the respondents stated that,

*There was so much support from WHO*, *including provision of a senior expert who basically run the show as there is no capacity in the MoH* (Ministry of Health official, Guinea).

The EU-Luxembourg-WHO programme went even further to render support for organisation of the consultation meetings, reorganisation of the ministry and the technical secretariat and financing of the dialogues. Some of the respondents felt that a context with that much support from a partner or donor would affect the negotiation power of the ministry of health and would be coerced to agree with the partner/donor.

Ideological conditions refer to the balance between a donor’s intent for the support and the ability of the country to factor that support in its vision [9]. The respondents from Liberia and Guinea lamented over the influence of donors in certain areas of the policy dialogues. Some of them believed that the limited capacity of ministry of health in the fragile context was a major factor in that influence. This type of dispositional power was also expressed in the interactions between the national and sub-national levels, characterised by directives on how and what to include in the sub-national dialogues.

Institutions, which evidently are weak in some of the five countries, are a factor in power relations. The institutional support required for the policy dialogue processes to take off included:

- Strengthening the dialogue structures, for example in Guinea;
- Commissioning several studies to build evidence on the areas for the dialogues, such as the studies on national health accounts in Liberia;
- Provide support for strengthening the health systems to the ministry of health, for example in Liberia and Guinea.

All these support needs reflect the institutional weaknesses of the ministry of health, which affect its negotiation capital and promote dependency on donors or partners for support. Knowledge and skills also were used as power agents with the invitation of experts to make presentations on specific topics during the policy dialogues. According to the respondents, the donor agencies invited and catered for these experts in most of the countries. One respondent in Liberia attested to this in this statement,

*In most of these dialogues we had experts from other countries who would come and present on various topics. For example if it is a topic of interest to a certain donor*, *they would ensure that they identify and fund the consultant* … *am not so sure if this is the right way of doing things* (National level official, Liberia).

From the perspective of power theories, this could be interpreted as a form of power exercise where donor partners with an interest in a specific area ensure that they have control over the speakers invited and, thus, have the upper hand over the issue of the dialogue. Knowledge has the power to shape peoples’ understanding and beliefs, which ultimately will determine their perspectives and decisions during the policy dialogues [9, 10].

Resources were an obvious source of dispositional power in many of the dialogues. The saying “he who pays the piper calls the tune” was applicable in several contexts in the dialogues. In reference to donor support, it was apparent that donors had a major role in the policy dialogues at the national level, as they financed the processes and provided and supported the research to generate the required evidence. They were also expected to support the activities that emanated from the dialogues.

Positional power as another form of dispositional power may be supported in organisations with the adoption of the common organisational philosophy of doing things the same way, sticking to known approaches and norms and resisting new approaches. The study found that some of the countries organised their dialogues through existing health forums with few alterations to the usual processes. These traditional meetings were characterised by presentations with little room for articulation of ideas and reflection, presentation of too many materials at the last minute, and lack of substantial data and evidence to support decision-making. In Chad concerns were raised that the dialogues were still conducted in the same manner as regular meetings, as one of the stakeholders stated,

*Yes*, *these are dialogues* … *however*, *the management is the same*, *late provision of reports*, *little time for in*-*depth discussions and understanding of the real issues*… *I doubt if there will be a lot of difference* (Donor partner, Chad).

This could be a form of control of the policy dialogues, which ideally should allow debate, interaction and discussion, but in cases such as these end up with only a few participants being given a chance to voice their concerns. Coupled with this was the lack of sufficient data, as noted in the interviews. Dialogues are an opportunity for far-reaching, realistic analysis, but in such contexts they tend to be driven by ideologies and perceptions [9]. Authors speaking about policy dialogues insist that for a dialogue to be fruitful it should include novel approaches for evidence sharing, timely distribution and discussion of background materials and proper facilitation for active participation and contributions [11, 12, 19, 20].

### Structural power

Structural power is concerned with how macro-societal structures shape and guide the conduct of individuals and agents [4]. Several examples from our study reveal how structural power was used to influence participant behaviour and manner during the policy dialogues. Structural power was used to rationalise the selection of certain topics for discussion in the dialogues, and it underpinned the legal and political conduct of the policy dialogues. For example, in Liberia it is cultural not to interrupt or interfere with someone who is speaking. This cultural practice was evident during the policy dialogue meetings. According to the respondents, some of the participants wasted a lot of time talking on irrelevant issues at the expense of others who might have wanted to talk. In both Guinea and Liberia there are good examples where political power was used during the Ebola outbreak to overshadow the role of the ministry of health. According to respondents, in these countries the president and other key political leaders took the centre stage during the Ebola outbreak. This had both positive and negative effects. On the positive side, their involvement made th**e** Ebola outbreak a priority, subsequently attracting the right resources. However less constructively, the technical role of the ministry of health, which was necessary to handle the outbreak, was undermined, as noted by one respondent,

*During the early days of the Ebola outbreak in Liberia*, *the Ministry of Health was powerless*, *as all the decisions and even the dialogues were overtaken by politicians and the presidency*. … *This contributed to the delay in halting the outbreak* (National NGO official, Liberia).

Legally, public officials in most of these countries are responsible and answerable to their superiors, following the hierarchy structure. This automatically poses a power challenge. There is a good example from Togo where policy dialogues were directed to begin at the grassroots level and feed into the upper levels.

In line with structural power, one of the common topics of issue was UHC. Although this was predetermined through the dialogue programmes, its legitimacy originated from a health equity and economic perspective. This example could also be regarded as relating to the use of knowledge power. A second example of economic structural power use relates to how the rationale for harmonisation and coordination emphasised through the dialogues was determined. Many of the stakeholders in all the five countries perceived the dialogues to be a means to enhance harmonisation and coordination of stakeholders leading to cost-effectiveness benefits. An example of this is depicted in the following quotation,

*I think these dialogues are helpful since were so disorganised. Every donor was going to the district and doing his own activities*… *now there is some control over what one does and if there is a duplicate this is sorted out*, *which is efficient* (Sub-national level official, Chad).

### Power, policy-making and context

Power is affected by the context, which is where the roles of the actors and their power can change and shift. One form of context is existing regimes. Much political literature suggests that African regimes can be broadly described as neo-patriotic. Such systems give prominence to relational power and influences, which tend to transcend bureaucratic, legal and administrative structures, as noted below,

*We at the sub*-*national levels have little say* … *We are often told to do several things since these dialogues began and sometimes they are contradictory*… *Of course you cannot question*, *you follow orders* (Sub-national level official, Guinea).

In this study, another form of context that affected the power dynamics was the macroeconomic situation, which is a form of structural power. In countries with weak and fragile economies the policy dialogues were dominated by the more economically powerful and wealthier stakeholders. Examples include the policy dialogues in Liberia and Guinea, where it was evident that a lot of support was given to the dialogues spearheaded by technical officers from WHO. Jones [21] suggests that there are three forms of influence on perspectives: evidence and advice, public campaign and advocacy, and lobbying and negotiations. There were several examples from our study where powerful stakeholders employed these strategies to influence perspectives and opinions, that could be regarded as use of power [21].

The declaration of the state of emergency during the Ebola outbreak in West Africa was a significant context in shaping the policy dialogues, rendering some actors powerless and others influential. In Liberia and Guinea, policy dialogues during the Ebola outbreak were taken over by non-health stakeholders and there was more involvement of community members in the policy dialogues than was the case previously. The community’s perceptions, beliefs and behaviours were central to halting the epidemic, and so they became important stakeholders, as noted below,

*The Ebola outbreak provided us with an important lesson about community involvement in the real sense. Without the communities we would not have halted the Ebola outbreak* (National level official, Liberia).

## Conclusion

Policy processes have often been regarded as linear and rational, but experience suggests otherwise. Policy dialogues, specifically are complex and irrational and involve many factors, one of which is power [9, 21]. Power is not always corrupt as its definition connotes. Power can have both positive and negative effects. Literature on power in policy dialogues argues that recognising and appreciating the different forms of power are important [1, 4, 9] as it provides a basis for using power in an effective manner during policy dialogues.

From our study we can confidently conclude that power has an important role to play in the policy dialogues. Using the Art and Tatenhoeve conceptual framework we were able to understand and appreciate the different forms of power and how they are used among actors. It was also clear that contexts such as emergency situations, macroeconomic circumstances and type of governance have a major influence on the dynamics of power among actors [4]. As seen in our study, power dynamics differed and changed depending on the context, an important example of which was the power dynamics during the Ebola outbreak, where some stakeholders lost their power to other more senior and political stakeholders. This is evident also from other studies where the context was shown to be a major influence in power dynamics [12, 13, 22].

One form of power is relational power, which can be either transitive or intransitive [4]. In our study transitional power was used on different occasions to benefit specific groups. Despite being considered to lead to a zero-sum game, in this context transitional power was used to facilitate changes in the dynamics of policy-making and discussion. The exercise of this power encouraged the government to think creatively and to contemplate some of the implementation issues often not regarded during policy-making. This form of power use should be encouraged. The civil society, other ministries, professional groups and unions should be invited to policy dialogues and provided with the opportunity to contribute to and negotiate for issues pertinent to specific policies.

Our study found that intransitive power was used in a positive manner to achieve the intended outcomes. Forums, meetings and formal groups worked in a unified manner to deliver on common outcomes. This is in line with what good policy dialogues are considered to be: participatory, debate-filled and engaging [14, 20].

There is a danger that the way transitional power is used might lead to doing things the usual way, without allowing room for innovative discussions and debate, as highlighted in some literature [9]. Dispositional power was the most complex form of power expressed both overtly and covertly. In this form of power, resources, knowledge and capacity have an influential role. It is also open to abuse if there are imbalances in negotiation capital among the actors [9]. In such instances the weaker actors can be easily coerced into decisions driven by powerful counterparts. There is a need to ensure that negotiation capital among actors is balanced for better policy dialogues. This can be achieved by building the capacity of actors to participate in policy dialogues. Capacity building can be in the areas of negotiation, policy influencing and persuasion [11, 15, 23]. However, policy dialogues should not be entirely dependent on knowledge and evidence. There is a tendency to favour the elite with evidence and knowledge, or what is referred to as technocratic policy-making [21].

Structural power is concerned with macro-societal structures that shape and guide the conduct of individuals and agents [4]. Our study found that all forms of this power were manifested during the policy dialogues. Cultural or social power was used to shape the manner of the participants, while legal structures were used to direct policy dialogues from the lower levels. Both structural and economic powers were exhibited to justify the discourses on UHC and harmonisation or coordination of partners. The right thing to do as a politician was demonstrated with the active participation of both Guinean and Liberian presidents during the devastating Ebola outbreaks.

Our study found that power was positively used during the dialogues to prioritise positive agendas, fast-track processes, reorganise positions, focus attention to details and involve communities. The negative effects of power during the dialogues included the use of position to control and shape the dialogues, using limited innovation, and influencing decisions and directions through the use of knowledge power. This study shows us that we need to be cognisant of the role of power during policy dialogues and that it is important to put in place mechanisms to control it effectively.

There is need for more research in this area to determine how to engender policy-making processes that are debate- filled and interactive through the positive use of power.

## Abbreviations

NGO, nongovernmental organisation; UHC, universal health coverage; WHO, world health organization
